# Reforming healthcare systems on a locally integrated basis: is there a potential for increasing collaborations in primary healthcare?

**DOI:** 10.1186/1472-6963-13-262

**Published:** 2013-07-08

**Authors:** Mylaine Breton, Raynald Pineault, Jean-Frédéric Levesque, Danièle Roberge, Roxane Borgès Da Silva, Alexandre Prud’homme

**Affiliations:** 1 Centre de recherche - Hôpital Charles Lemoyne, Longueuil, QC, Canada; 2 Université de Sherbrooke, Sherbrooke, QC, Canada; 3 Institut national de santé publique du Québec, Montreal, QC, Canada; 4 Centre de recherche du Centre hospitalier de l’Université de Montréal, Montréal, QC, Canada; 5 Direction de santé publique de l’Agence de la santé et des services sociaux de Montréal, Montréal, QC, Canada; 6 Université de Montréal, Montréal, QC, Canada

**Keywords:** Primary care, Network, Inter-organization collaboration

## Abstract

**Background:**

Over the past decade, in the province of Quebec, Canada, the government has initiated two consecutive reforms. These have created a new type of primary healthcare – family medicine groups (FMGs) – and have established 95 geographically defined local health networks (LHNs) across the province. A key goal of these reforms was to improve collaboration among healthcare organizations. The objective of the paper is to analyze the impact of these reforms on the development of collaborations among primary healthcare practices and between these organisations and hospitals both within and outside administrative boundaries of the local health networks.

**Methods:**

We surveyed 297 primary healthcare practices in 23 LHNs in Quebec’s two most populated regions (Montreal & Monteregie) in 2005 and 2010. We characterized collaborations by measuring primary healthcare practices’ formal or informal arrangements among themselves or with hospitals for different activities. These collaborations were measured based on the percentage of clinics that identified at least one collaborative activity with another organization within or outside of their local health network. We created measures of collaboration for different types of primary healthcare practices: first- and second-generation FMGs, network clinics, local community services centres (CLSCs) and private medical clinics. We compared their situations in 2005 and in 2010 to observe their evolution.

**Results:**

Our results showed different patterns of evolution in inter-organizational collaboration among different types of primary healthcare practices. The local health network reform appears to have had an impact on *territorializing* collaborations firstly by significantly reducing collaborations outside LHNs areas for all types of primary healthcare practices, including new type of primary healthcare and CLSCs, and secondly by improving collaborations among healthcare organizations within LHNs areas for all organizations. This is with the exception of private medical clinics, where collaborations decreased both outside and within LHNs.

**Conclusion:**

Health system reforms aimed at creating geographically based networks influenced primary healthcare practices’ both among themselves (horizontal collaborations) and with hospitals (vertical collaborations). There is evidence of increased collaborations within defined geographic areas, particularly among new type of primary healthcare.

## Background

Improving collaboration among healthcare organizations has been a policy goal in numerous countries over the past decade [1-3]. For example, since 1972, Finland has had a primary health care system based on health centres run and funded by the local munipalities [4]. Almost 70% of health centres have primary healthcare services and social services within the same administrative governance. About 20–30 of the health organizations combine primary care and secondary care [4]. In England, *Care Trusts*, an organisation based on primary care trusts and primary care groups, are responsible for the commissioning and provision of all local health and social care [5]. In this paper, we analyze the impact of major public healthcare system reforms in the Canadian province of Quebec in terms of collaboration among healthcare organizations.

### Historical background

Quebec is a province of over 8 million residents with a tax-based system providing universal access to medical services. Healthcare organizations, such as community health centres and hospitals, receive block funding from the Ministry of Health and Social Services, and physicians are remunerated predominantly on a fee-for-services basis. While nearly all family physicians provide medical services reimbursed by the public Medicare program, most primary healthcare practices are private rather than public enterprises with regards to their ownership. The responsibility for organizing primary healthcare services has historically been left to these community-based private medical practices owned by a physician or a group of physicians. This situation stands in sharp contrast to that of other healthcare organizations, such as hospitals and community health centres, which form an integral part of the public system. Until quite recently, although physicians were reimbursed by the public health insurance system for services [6] there had been very little public investment in primary healthcare services provided in private practices. Delivery of primary healthcare was at the periphery of the system rather than at its core [7].

Historically, private medical clinics have been the dominant type of primary healthcare in Quebec, and these had established very few links with public healthcare organizations. In the early 1970s, the government launched an ambitious new type of public primary healthcare provision by introducing local community services centres (CLSCs). These primary healthcare practices were entirely public, in terms of funding, infrastructure and resources, as well as governance. CLSCs were particularly innovative with regard to governance firstly because it was under the hierarchical responsibility of the Ministry of Health and Social Services (MSSS) and also because it incorporated a social services component into the provision of healthcare services [6]. A variety of professionals including physicians, nurses, occupational therapists, physiotherapists, nutritionists, psychologists and social workers work in CLSCs which provide both preventive and curative services, as well as support services such as home care. Originally, CLSCs were meant to become the main entry point into the healthcare system. However, physicians’ associations vehemently opposed the practice conditions associated with this innovation, particularly the fact that CLSC physicians were salaried. Few family physicians (20%) elected to practice in these facilities and only a small proportion of the population identifies them as the source of primary healthcare services [8]. Thus, CLSCs have been consigned to minority status with regard to their coverage of the population’s medical needs. At the same time that CLSCs were being implemented, a network of privately owned practices developed rapidly, supported by Quebec’s association of general practitioners, the *Fédération des médecins omnipraticiens du Québec*, but without any direct control of their activities from the State [9]. By 2000, Quebec’s primary healthcare network was made up of 147 CLSCs and 800 private medical clinics.

Given the CLSCs’ relative failure to attract physicians and the limitations of primary healthcare services organization at that time, the Clair Commission in 2000 proposed a new type of primary healthcare practice, the Family Medicine Group (FMG), to improve healthcare services’ organization and delivery [10]. Based on contractual agreements with the provincial government [7], FMGs consist of a group of physicians working in close collaboration with nurses to provide services to registered patients. Unlike CLSC, the establishment of FMGs does not require the creation of new structures because they are grafted onto existing organizations [11]. The majority of FMGs are privately owned organizations. The FMG reform makes provisions for the recruitment of nurses and administration staff, and the acquisition of informatics equipment. On average, one FMG serves around 15,000 people and has around ten physicians, two nurses, and two administration support staff. These measures represent a budget of nearly $200 000 CAN for infrastructure in exchange for compliance with certain conditions such as services provided Monday to Friday, with and without appointment. On weekends and holidays, a minimal level of walk-in services must be available [12]. Since the FMG policy was inaugurated in 2002, the number of accredited practices has steadily increased [6]. As of November 2012, there were 250 accredited FMGs in Quebec, enrolling over 30% of the province’s population.

Recently, in response to problems pertaining to accessible healthcare, another type of primary healthcare was put forward: network clinics. This type of practice is also grafted on existing organizations. They are larger private clinics than those that are eligible to become GMF. Network clinics had been established in many regions through contractual agreements with regional health authorities [7]. Network clinics are large privately owned group practices providing extended hours of clinical services. Services are provided seven days a week and network clinics have on-site access to extended diagnostic services such as imagery and laboratory testing.

**Figure 1:**
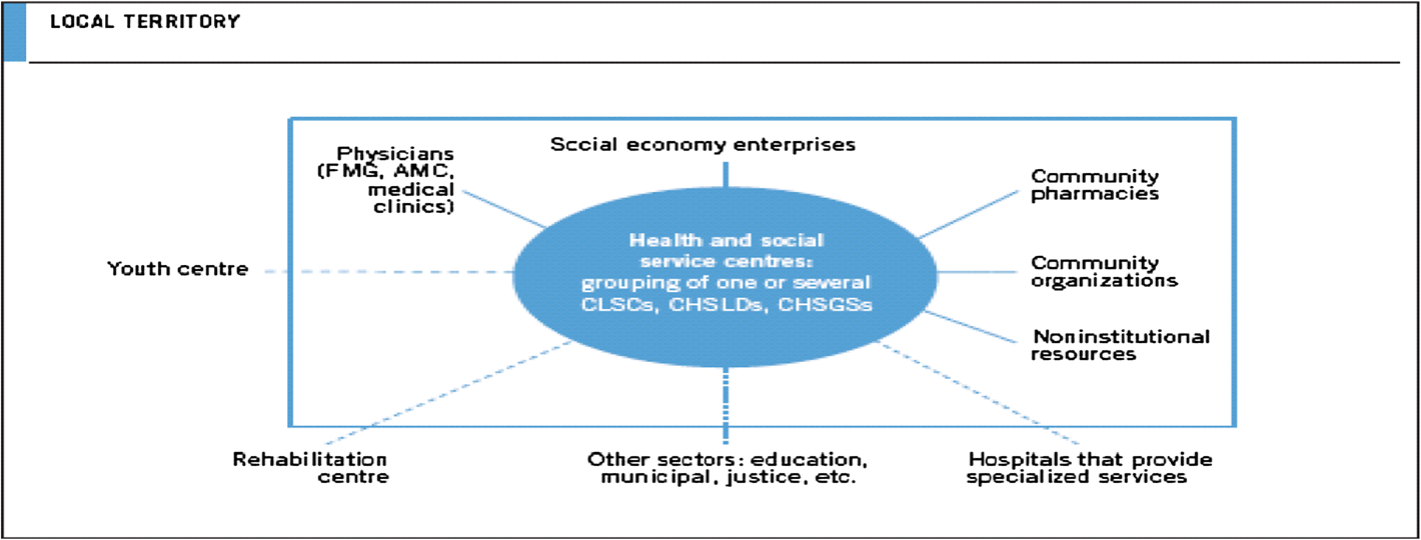
The local health network.

In addition to these primary healthcare practice reforms, in 2004 the Quebec government initiated a large-scale redesign of its health system structure with the objective of improving accessibility, continuity, integration and quality of services for the population of a given area, by setting up integrated local health networks (LHNs) across the province. At the heart of the local health networks, 95 new organizations, called Health and Social Services Centres, were created by merging territorially-based CLSCs with long-term care institutions and, in 85% of cases, an acute care hospital [13]. The merger of all these organizations to form Health and Social Services Centres is illustrated in the box at the centre of Figure 1. Each Health and Social Services Centre was formally mandated to lead the creation of a local health network (LHN) by encouraging the establishment of formal or informal arrangements among various providers within its territory presently offering services [14]. These LHNs were largely created through virtual integration in the form of alliances and partnerships among autonomous organizations at different levels of care.

The creation of Health and Social Services Centres are seen as a means for bringing inter organizational collaboration and integration of health services [15]. They have to establish agreements with private medical clinics, which had never been well integrated previously. The participation of family physicians in planning and organizing primary healthcare services was initially disappointing [16]. It is only recently, with the creation of Health and Social Services Centres that we recognize the network of private clinics as an important and indispensable partner in efforts to reconfigure primary healthcare services [17].

This study followed the launch of two consecutive reform policy initiatives from Quebec’s Ministry of Health and Social Services: the creation of new type of primary healthcare such as FMGs and network clinics as well as the establishment of LHNs under the governance of Health and Social Services Centres. New type of primary healthcare were implemented to increase accessibility and continuity of care, while Health and Social Services Centres aimed at better coordinating and integrating services by creating territorially-defined LHNs. The structural integration of all CLSCs, and in most cases hospitals, into Health and Social Services Centres through a merger process aimed at facilitating collaboration among these organizations under a single governance structure. The new Health and Social Services Centres were in turn required to develop contractual agreements with other providers within their territories offering services essential to the local population, thus creating a local health network. Although the policies were proposed respectively in 2002 and 2004, implementation largely began in 2005, coinciding with the first data collection of our study. Five years later both reforms were well established and, based on a second data collection, we were able to analyze how the evolution of primary healthcare practices had translated into measurable effects on inter-organizational collaborations.

### Theoretical background

Many countries have experimented with different organizational structures and processes to facilitate better collaboration among providers, improve coordination of services between organizations, and provide more integrated healthcare to populations. These countries have attempted to apply the principles of integrated care to health reforms as a potential solution to many long-standing problems [18-20] and are thus moving toward more integrated healthcare.

Since the seminal work of Shortell et al. [20], the definitions and models of integrated healthcare have focused on coordination of health services across the continuum of care as well as collaboration among providers and organizations in delivering of services [21-23].In such a way, strengthening links between providers and healthcare organizations has been a policy goal for numerous countries over the past decade [1-3]. Improving inter-organizational collaborations remains vital because the magnitude of health problems is such that any single organization is easily overwhelmed. Need for services typically crosses organizational boundaries and resources are constrained [24]. In this paper, collaboration between organizations of a similar level of care, such as primary healthcare practices, is labelled *horizontal collaboration*, and collaboration between organizations of different levels of care, such as primary healthcare practices and hospitals, is labelled *vertical collaboration*[20]. Few studies have analyzed collaborations among primary healthcare practices [1,2,24] in this way.

Researchers have paid even less attention to assessing the impacts of reform policies on emerging primary healthcare models and of LHNs on inter-organizational collaborations [25]. We hypothesize that the combination of two major reforms has had a synergistic effect on inter-organizational collaboration. The creation of LHNs was expected to “territorialize” collaboration, by strengthening partnerships among organizations within the local health network whilst at the same time, decreasing the number of relationships outside the local health network. FMGs and network clinics should be integrated into communities, which should in turn encourage the development of relationships with other healthcare organizations within the local health network.

The objective of this paper is to analyze the impact of the Quebec reforms on the development of collaborations among primary healthcare practices and between these organisations and hospitals, both within and outside the geographically defined local health networks, by comparing collaboration levels at two points in time so as to observe evolution. In the following sections, we present our methodology and the results obtained. We then discuss these results, looking particularly at the influence of primary healthcare types. We conclude with recommendations to guide future policies aimed at increasing collaboration in healthcare systems.

## Methods

### Data sources

Our level of analysis was primary healthcare practices. We used data from two organizational surveys we conducted in 2005 and 2010 in Quebec’s two most populous regions, Montreal and Montérégie, where a survey was mailed to the leader of each medical practice in the 23 LHNs under study. The study protocol was approved by the *Agence de la santé et des services sociaux de Montréal* Ethics committee, and participation was voluntary. In 2005, we contacted all 659 existing medical clinics; in 2010, that number was 606, all of whom were contacted. The response rates were 72% in 2005 (473 clinics) and 62% in 2010 (376 clinics). To analyze the evolution of collaborations, we used only the data from the surveys of those primary healthcare practices (n = 297) that responded to surveys in both 2005 and 2010. The combined response rate was thus 55% (n = 541 clinics that had existed in both survey instances). Table 1 shows the different types of primary healthcare practices in 2010 that had responded to both surveys. Table 1 also shows the proportion of the population that had identified, the different types of primary healthcare practices as a regular source of care. This data is from a population survey we had undertaken in conjunction and linked with the practice surveys, as part of a broader study of people’s utilization and experience with primary healthcare services.

**Table 1:** Comparison of primary healthcare practices that existed in 2005 and 2010 and responded to both surveys

**Type of primary healthcare organization**	**Sample (n = 297)**	**Population covered*(C.I. 95%)***
First-generation FMG	13.5% (n = 40)	23.9% *(22.9% ; 24.9%)*
Second-generation FMG	9.4% (n = 28)	11.2% *(10.4% ; 12.0%)*
Network clinic	6.7% (n = 20)	15.1% *(14.2% ; 16.0%)*
CLSC	10.8% (n = 32)	4.6% *(4.1% ; 5.1%)*
Private medical clinic	59,6% (n = 177)	45.1% *(43.9% ; 46.3%)*

Half of the population (almost 50%) identified private medical practices as their regular source of care. These practices are not associated with a new model of primary healthcare; they comprised almost 60% of the organizations responding to our surveys. First-generation FMGs are defined as clinics that were accredited as FMGs before our first data collection in 2005, while second-generation FMGs are those that were accredited after 2005 and before our second data collection. First-generation FMGs comprised almost 14% of the organizations responding to our surveys, and second-generation FMGs, comprised almost 10%. First-generation FMGs were identified as a regular source of care by 24% of the population, compared with almost 12% for second-generation FMGs. The first network clinics were instated in 2005, after our first data collection, therefore there is no first generation network clinics in our study. They represent 6% of the primary healthcare practices responding to our survey and cover almost 14% of the population. Finally, while CLSCs constituted almost 10% of organizations responding to our surveys, their population coverage is marginal, with less than 5% of population consulting them for medical care. Implanted in the early 1970s, these defined Quebec’s first type of primary healthcare, and were all eventually merged into Health and Social Services Centres, within which they continue to operate.

We categorized horizontal and vertical collaborations by measuring any formal and informal arrangements reported by respondents with other primary healthcare practices or hospitals, for various types of activities such as services planning, access to diagnostic services such as radiology and laboratories, pooling of resources, as well as patient referral or follow-up. Table 2 shows the questions addressed to the directors of each medical clinic characterizing the different kinds of collaboration.

**Table 2:** Organizational survey questions to primary healthcare organization

Collaboration with PHC organization(s)	**Indicate whether your clinic has formal or informal arrangements with one or several PHC medical clinic(s) for the following activities:**
	Planning services offer (on-call activities, clinic hours, walk-in services, etc.)?
	Access to technical services (e.g. radiology, laboratory)?
	Exchange resources (e.g. loan of professionals)?
	Referral or transfer of patients to general practitioners, specialists or other professionals?
	Follow-up for hospitalized patients or patients seen at the clinic?
	**If you answered “yes” to any of the choices in the preceding question, identify the main primary healthcare clinic or clinics with which you have arrangements.**
Collaboration with hospital(s)	**Indicate whether your clinic has formal or informal arrangements with one or several hospital(s) for the following activities:**
	Planning services offer (on call activities, clinic hours, walk-in services, etc.)?
	Access to technical services (e.g. radiology, laboratory)?
	Exchange resources (e.g. loan of professionals)?
	Referral or transfer of patients to family physicians, specialists or other professionals?
	Follow-up for hospitalized patients or patients seen at the clinic?
	**If you answered “yes” to any of the choices in the preceding question, identify the main hospital or hospitals with which you have arrangements.**

### Data analysis

Horizontal or vertical collaboration was measured based on the percentage of clinics that reported having participated in at least one activity with another organization (collaboration score). We categorized collaborations as being within or outside the local health network territory based on the location of the organization identified by the respondents. We used a database of all the medical clinics of the two regions under study to determine their locations. The scores for each type of primary healthcare organization in 2005 and 2010 were compared using paired-samples t-tests. All analyses were conducted using Predictive Analytics Software (PASW version 18).

## Results

**Figure 2:**
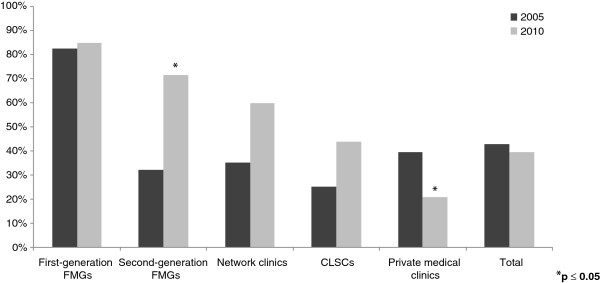
Horizontal collaborations within local health networks.

**Figure 3:**
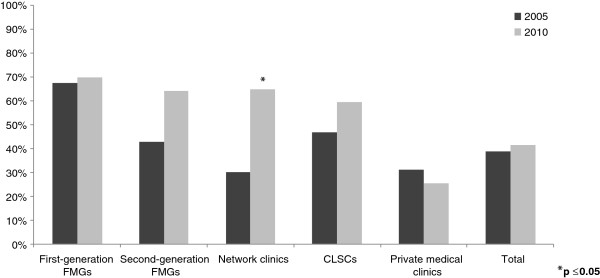
Vertical collaborations within local health networks.

**Figure 4:**
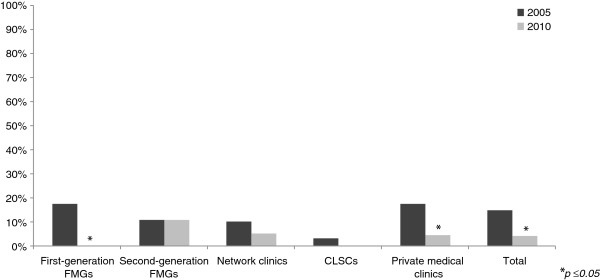
Horizontal collaborations outside local health network.

**Figure 5:**
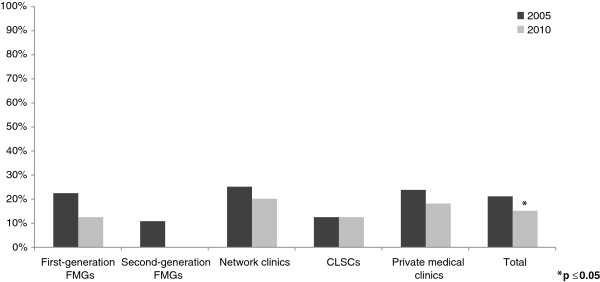
Vertical collaborations outside local health network.

After the launch of these two major reforms in Quebec, several changes in inter-organizational collaborations were observed between 2005 and 2010. Figures 2, 3, 4, 5 present the evolution of the proportion of clinics that reported having at least one formal or informal arrangement with another organization within or outside their local health network. Descriptive statistics in support of Figures 2, 3, 4, 5 are presented in Additional file 1.

### Collaborations within local health networks

One goal of the reform was to strengthen collaboration among organizations within local health networks. As seen in Figure 2, the average level of collaboration among primary healthcare practices within a local health network decreased slightly overall, but not significantly, from 42.8% in 2005 to 39.4% in 2010. However, with regard to the impact of the new type of primary healthcare, our results showed different patterns of evolution. For clinics already accredited as FMGs in 2005, whose level of horizontal collaboration within LHNs was already considerable, collaboration levels did not change in 2010 (82.9%). Clinics accredited during our five-year analysis period showed considerable improvement in terms of collaborations. Second-generation FMGs increased collaboration within their local health network significantly, from 31.0% in 2005 (before they were accredited) to 75.9% in 2010, and network clinics increased from 38.9% in 2005 to 55.6% in 2010. CLSCs’ collaboration levels also increased from 22.6% to 45.2%. We noted a significant decrease in horizontal collaboration within LHNs for private medical clinics, from almost 40% to 20%.

As seen in Figure 3, primary healthcare practices’ overall average collaboration with hospitals within their local health network increased slightly but not significantly, from 38.7% to 41.4%. However, among the different types of primary healthcare practices, medical clinics accredited as FMGs or network clinics after 2005 significantly improved their vertical collaborations with hospitals within their local health network, from 41.4% to 69.0% for second-generation FMGs, and from 33.3% to 61.1% for network clinics. For FMGs already accredited in 2005, we did not observe significant change; they were already high in 2005 and were highest in 2010, at 70.7%. CLSCs also increased from 45.2% to 58.1%. For private medical clinics, vertical collaborations decreased slightly, from 30.9% to 25.3%.

### Collaborations outside local health network

Overall, collaboration with organizations outside the local health networks’ areas decreased significantly. As seen in Figure 4, primary healthcare practices’ overall average collaboration with other primary healthcare practices outside their area decreased significantly, from 14.8% in 2005 to 4.0% in 2010. Among the different types of primary healthcare practices, the patterns of evolution all went in the same direction; collaborations outside LHNs decreased over time, except for second-generation FMGs, where no change was observed. We observed the most significant change in FMGs already accredited in 2005, from 17.1% in 2005 to 0% in 2010. Also, private medical clinics, which represented the majority of the clinics, significantly decreased collaboration with other clinics outside their local health network, from almost 17% to less than 4%.

Finally, as seen in Figure 5, primary healthcare practices’ overall average collaboration with hospitals outside their local health network decreased, from 21.2% in 2005 to 15.2% in 2010. Of the different types of primary healthcare practices, second-generation FMGs and CLSCs did not change over time, while the others decreased slightly. First-generation FMGs and network clinics decreased more over time compared with private medical clinics. In 2010, FMGs had, at most, 12% of vertical collaborations outside local health networks, as compared with private medical clinics, which had almost 18%. Network clinics scored highest, with 22% vertical collaborations outside their LHNsin 2010.

### Nature of collaboration

Table 3 shows the evolution between 2005 and 2010 of the nature of vertical and horizontal collaborations among type of primary healthcare practices. The details of the nature of collaboration among primary healthcare practices show that the main collaboration occurs in the area of planning health services. In this kind of collaboration, second generation FMGs improved significantly, from 10% in 2005 to more than 75% collaboration in 2010 whilst network clinics improved less, from 20% to 40%. However, a surprising result is that CLSCs did not improve a lot, from 22% to 31%, even if they were all merged into the Health and Social Services Centres that received formal mandates pertaining to coordinating the planning and organizing of the local health network.

**Table 3:** Details of the comparison among different model of primary health care for different type of collaborations

**Horizontal collaboration : Planning services offered (%)**					
	**2005**	**2010**	**df**	**T-value**	**P-value**
First-generation FMGs	77,5%	82,5%	39	−0,628	0,534
Second-generation FMGs	10,7%	75,0%	27	−6,971	0,000
Network clinics	20,0%	40,0%	19	−1,453	0,163
CLSCs	21,9%	31,2%	31	−0,828	0,414
Private medical clinics	20,9%	11,9%	176	2,244	0,026
Total	27,6%	31,3%	296	−1,095	0,274
**Horizontal collaboration : Exchange of resources (%)**					
	**2005**	**2010**	**df**	**T-value**	**P-value**
First-generation FMGs	17,5%	32,5%	39	−1,778	0,083
Second-generation FMGs	3,6%	10,7%	27	−1,000	0,326
Network clinics	5,0%	15,0%	19	−1,453	0,163
CLSCs	6,2%	15,6%	31	−1,791	0,083
Private medical clinics	4,5%	2,8%	176	1,000	0,319
Total	6,4%	9,8%	296	−1,833	0,068
**Vertical collaboration : Access to technical services (%)**					
	**2005**	**2010**	**df**	**T-value**	**P-value**
First-generation FMGs	47,5%	52,5%	39	−0,530	0,599
Second-generation FMGs	25,0%	32,1%	27	−0,570	0,573
Network clinics	35,0%	70,0%	19	−2,333	0,031
CLSCs	37,5%	53,1%	31	−1,305	0,201
Private medical clinics	22,6%	19,8%	176	0,728	0,467
Total	28,6%	32,3%	296	−1,106	0,270
**Vertical collaboration : follow-up for hospitalised patients or patients seen at the clinic (%)**					
	**2005**	**2010**	**df**	**T-value**	**P-value**
First-generation FMGs	67,5%	57,5%	39	0,941	0,352
Second-generation FMGs	42,9%	53,6%	27	−0,721	0,477
Network clinics	40,0%	40,0%	19	0,000	1,000
CLSCs	37,5%	31,2%	31	0,701	0,488
Private medical clinics	32,2%	22,0%	176	2,534	0,012
Total	39,1%	32,0%	296	2,061	0,040

The second main collaboration occurs related to exchange of resources. The first generation FMG improves the most in this kind of collaboration, from 18% in 2005 to 32% in 2010. For the others types of primary healthcare practices, there was very little collaboration of that kind, around 5% and it improved to slightly less than 15% in 2010. This is with the exception of private medical clinic that decreased less than 3%. This result suggests that primary healthcare practices need a longer period of time to develop collaborations pertaining to sharing resources.

The details of the nature of collaboration among primary healthcare practices and hospital show that the main collaboration occurs regarding access to technical services such as radiology and laboratory. Network clinics improved the most in this kind of collaboration -from 35% in 2005 to almost 70% of collaboration in 2010 with a hospital. Except private medical clinics, all other types of primary healthcare practices improve on access to technical services. On follow-up for hospitalised patients, all type of primary healthcare decrease on that kind of collaborations. Except for private medical clinics, those results were not significant.

## Discussion

### Reforming healthcare systems on a locally integrated basis: is there potential for increasing collaborations in primary healthcare?

The Health and Social Services Centres’ mandate to lead the creation of local health networks re-oriented services organization toward a more territory-based perspective [17]. The philosophy underlying the new service delivery models transformed the Health and Social Services Centres’ organizational perspective into one that was more territorial and population-based by strengthening links among healthcare organizations within the local health networks’ boundaries. Recent evaluations of the reform, which was designed to integrate all the health and social care services in each geographical territory, have shown that the various institutions of the healthcare system are indeed becoming more integrated [14,26]. Thus, a territory-based vision has gradually emerged [17]. The philosophy that sustained the development of services gradually transcended Health and Social Services Centres’ organizational boundaries as managers started to collaborate with other organizations, such as medical clinics in their area [27]. Several examples were observed regarding the development of collaborations such as negotiation with hospitals for privileged access to technical support for primary healthcare organization, referrals for patients without family physicians and support for medical clinics during their accreditation process as FMG or Network clinic. According to a recent study, private medical clinics and Health and Social Services Centres were at the beginning a establishing a dialogue and becoming acquainted with each other. That represents a major step of the reform [17].

Our results provide more specific information on the nature of these inter-organization collaborations. For horizontal collaborations, our results suggest different patterns of evolution in the different types of primary healthcare practices. For first-generation FMGs, the local health network reform did not significantly improve their collaborations with other primary healthcare practices, as their scores for collaboration within the local health network were already the highest. However, for clinics adopting a new type of primary healthcare during the implementation of the local health network reform, such as FMGs and network clinics, our results showed a major improvement in horizontal collaborations. We might argue that the clinics established earlier, which were the early adopters, were more receptive to change and more sensitive to the context of reform. They acted as leaders in the field and later on they had little room left for improvement because they had already established a high level of collaboration. In this way, we observed a levelling-off effect over time. Clinics that obtained their FMG or network clinic accreditation after 2005 had more room for improvement; hence it is not surprising they recorded more change over time. However, organizational change takes time, and we can expect that inter-organizational collaborations will continue to improve in that group to potentially attain the same levels of collaboration as the first generation FMGs. We observed what appeared to be a gradual mimetic effect on other primary healthcare practices in general. Almost 15% of the clinics not accredited in 2005 became second-generation FMGs and network clinics between 2005 and 2010. The increase in clinics adopting the new type of primary healthcare suggests movement in the direction of the local health network reform goal of extending inter-organizational collaborations among primary healthcare practices.

The main type of horizontal collaboration occurs in planning of health services. Our results show that CLSCs appear more involved in social services and do not participate as much in planning of medical services. This may withdrawal of CLSC from the field of medical services thus enhancing their specific primary role in social services. When it comes to sharing resources, primary healthcare practices are much more reluctant to engage further in real partnership and collaborations. Studies over time would reveal where this constraint can be overcome.

In terms of vertical collaborations, the results highlight the same patterns of evolution as seen for horizontal collaborations. However, based on the collaboration scores, our results suggest a smaller influence of local health network reform on vertical collaborations than on horizontal collaborations. The details of the nature of collaboration among primary healthcare practices and hospital show that the main collaboration occurs regarding access to technical services. Network clinics improved the most in this type of collaboration. This result is not surprising because network clinics’ priority is to enhance access to technical services. Almost 70% of the network clinics had on-site access to extended diagnostic services. That may also explain why around 30% of network clinics did not have formal or informal collaboration with a hospital. The second kind of collaboration with hospitals regards the follow-up of hospitalized patients. This kind of collaboration did not improve significantly. The result is more surprising for CLSCs because Health and Social Services Centres and hospitals are under the same governance structures. One major argument behind the merger was that new structure would facilitate the coordination among health organizations. Our results did not support this hypothesis. This could be the result of a stronger emphasis of merged organisations in dealing with their acute and long-term hospital-based services more than an actual integration of their community-based services.

Overall, the local health network reform has had a “territorializing” effect on collaborations; by decreasing the organizations’ relationships with primary healthcare practices and hospitals outside their local health network and by strengthening the development of collaborations among health organizations within their local health network. Again, private medical clinics were the exception. Clearly, merging the planning and provision of healthcare services on an area-based perspective can work better if a parallel investment in organising primary-care practices is made. The contractual arrangements which are intrinsic to the current reform of primary healthcare in the context of our study could be a strong contributor. Such contractual arrangements remain absent in private practices.

### Increasing collaboration in a context of isolated primary healthcare practice: challenges ahead?

Although we observed significant improvement toward meeting the reform’s goals in clinics that had adopted new type of primary healthcare, the strategy of changing the system incrementally through voluntary participation produced only a marginal improvement of inter-organizational collaboration overall. This local and targeted strategy seemed to have had a limited impact at the broader healthcare system level. Our results suggested that a majority of clinics remained untouched by the current reform and were experiencing a decrease in collaboration. Independent physician-owned and managed clinics and small group practices, which predominate in the system, seemed to have withdrawn and disengaged from activities organized at the local health network level.

The mandatory creation of LHNs was not associated with any mandatory reform of primary healthcare practices. The emergence of new types of primary healthcare occurred on a voluntary basis. The important increase regarding within local health network collaborations among clinics accredited under new type of primary healthcare, and the parallel decrease among organizations that were not part of a reform model, could suggest that the reform was preferentially benefiting new models at the expense of non-reform models. However, this would need to be tested with further research.

In terms of implementing the new types of primary healthcare, the reform is still incomplete, and the remaining clinics with no accredited status may be more difficult to reach using a very rigid prescriptive model. The criteria for accrediting FMGs or network clinics currently limit the implementation of new FMGs or network clinics in clinics where large groups of physicians practise. This excludes the majority of clinics with smaller physician groups. Based on administrative data, we know that almost 60% of the private medical practices had fewer than three practising physicians [28]. The next step could involve broadening the accreditation criteria to allow for a new type of primary healthcare based on “softer” criteria that would encourage these smaller clinics to form networks providing a defined range of services, in exchange for incentives, such as human, material, and financial resources, currently offered to FMGs.

### Improving collaborations: the impact of professional roles, technological support and governance mechanisms

As mentioned, our results suggested that the different types of primary healthcare practices showed different patterns of evolution in collaborations. The development of collaboration has been facilitated by the implementation of new type of primary healthcare such as FMGs and network clinics. Some Health and Social Services Centres actively supported establishment of the new type of primary healthcare as a way to improve the delivery and integration of services offered to the population of their territory [14]. These were associated with contractual agreements between accredited clinics and other healthcare institutions at the local level to provide a defined range of services. These contractual agreements formalized the collaborations and the sharing of resources among and within primary healthcare clinics. In exchange for becoming accredited, medical groups have benefited from added human, material, and financial resources. For example, the introduction of nurses into FMGs has produced real changes in medical practice in private clinics by fostering closer collaboration between physicians and nurses. Also, nurses assigned to an FMG maintain an employment link with the Health and Social Services Centres, but are under the functional authority of the FMG. Maintaining the nurses’ institutional links creates more formal alliances and encourages collaboration among FMGs and between these and other healthcare organizations in the local health network [6].

A major barrier to coordination of care among organizations is the gap in clinical information sharing and a significant lag in the use of information technologies [29]. Quebec has been particularly slow to implement electronic technology, which is fundamental for professionals’ clinical practices and communication with partners [7,30]. However, FMGs have been aided in establishing electronic medical records and other tools linking the practices with hospitals (lab test results, electronic prescribing, etc.), which could explain the increase for within local health network collaboration associated with FMG models.

### Strengths and limitations

Our study analyzed the impact of policy reforms on increasing collaboration among healthcare organizations within local health networks. The strength of our study is that the data for analyzing the impact of the reforms comes from two system-wide assessments of primary healthcare practices, done five years apart, with a large number of primary healthcare practices undergoing a sort of natural experiment.

However, several limitations should be noted. First, our research was set in a specific organizational context, the province of Quebec. Québec healthcare system’s is defined by universal access to medical services. The majority of primary healthcare is privately owned and family physicians are paid on a fee-for-services basis. Thus, this specific context may limit the generalization of our results in other contexts.

A second limitation pertains to the fact that our scope of analysis is only five years, and reforms take time. Although the design enables comparison of the same organizations at two points in time, it does not allow for comparison with a control group where the reform has not been implemented. A further limitation is that our sample represented approximately 55% of the clinics, and the new types of primary healthcare were over-represented. Furthermore, as with any study using respondents’ perceptions, this study could suffer from perception bias and desirability bias. Respondents could give a biased portrait of their organization’s characteristics. Another important limitation is that the respondent to the questionnaire may have been different in some practices in 2005 and 2010, thus introducing a possible respondent’s bias. This would however create a conservative bias since more positive respondent could have been present in 2005 and in 2010 and therefore create a regression to the mean and reduce the capacity of this study to find significant differences.

Also, some potential cofounder variables could have influenced the evolution of collaborations over time. A lot of studies on primary healthcare suggest that the context of the implementation such as in rural or urban settings may influence the collaborations among organizations [31,32]. One argument behind this is that it is easier to establish local health network involving less participants. In our study, 32 primary health care organizations were located in rural settings and 265 primary healthcare practices were located in urban settings. When we compared those two groups, we found more collaboration among primary healthcare practices located in rural area. However, the evolution of change over 2005 and 2010 remain similar among the two groups. See Additional file 2 for more details on the comparison among clinics located in urban areas and clinics located in rural areas.

Furthermore, the role played by some contextual elements is known to have influence on the dynamics of the establishment of collaboration [33-36]. For example, the roles played by Health and Social Services Centres in the creation of local health network influenced the evolution of collaborations and we did not control for that.

The collaboration measures were constructed using only two kinds of healthcare organizations: primary healthcare practices and hospitals. We did not have data on collaborations with other types of healthcare organizations such as pharmacies, specialist clinics or community organizations. This limitation would tend to underestimate inter-organizational collaboration. In addition, the thresholds identified to measure inter-organizational collaboration were the lowest possible. To construct the measure we used the minimal collaboration occurring between organizations for at least one type of activity. We did not assess the intensity of the collaboration.

Also, our results may have slightly underestimated the impact of the local health network reform on vertical collaborations outside LHNs due to the fact that three of the 23 LHNs studied had no hospitals within their territory. When we removed from the scoring the 24 medical clinics (8% of our sample) located in those three local health networks, “average vertical collaborations outside local health network” decreased from 19% in 2005 to 13% in 2012, compared to 21% to 15% when those 24 clinics were included. Network clinics located in territories with no hospital (n = 2) had the highest rate of outside local health network - vertical collaboration. When we removed those two network clinics from the measure of vertical collaboration outside local health networks, the score for network clinics was 12%, compared to 22% when those two network clinics were included in the scoring. Network clinics depend on advanced access to specialized care. Thus, it is not surprising that network clinics in LHNs without a hospital created arrangements with hospitals outside their geographic boundaries. However, our results suggested that for network clinics located within a local health network that included a hospital, arrangements with hospitals outside their local health network were limited, even for LHNs in an urban territory. The presence of those 24 clinics in the three LHNs without a hospital did not affect the measurement of the evolution of vertical collaborations within local health networks, since no improvement could be observed over time in terms of collaboration with hospitals within their local health network.

## Conclusion

The literature suggests that strong primary healthcare is an essential foundation for successful healthcare system integration [30,37]. Reforms have been implemented in several different jurisdictions for emerging innovations in primary healthcare models and to encourage relationships among primary healthcare practices. We analyzed two consecutive reform policy initiatives in Quebec: the creation of new primary healthcare models and the establishment of local health networks. Our results showed that both policies seem to have had an impact on strengthening inter-organizational collaboration. The mandated creation of LHNs improved inter-organizational collaborations within LHNs for new type of primary healthcare, while collaboration appeared to have diminished in older medical clinic type. Our results suggested that the local health network reform has had a positive effect on territorializing collaboration by significantly reducing collaborations outside LHNs and, less significantly, for the emerging new type of primary healthcare and CLSCs, by improving collaboration among health organizations within the local health networks. Our results showed that the new type of primary healthcare seemed to have contributed more to increasing inter-organizational collaborations than did other types of clinics.

## Competing interests

The authors declare that there are no competing financial or non financial interests.

## Authors’ contributions

MB performed the statistical analysis and drafted the manuscript. RP conceived the study, participated in its design and coordinated and helped to draft the manuscript. JFL also conceived the study, participated in its design and coordinated and helped to draft the manuscript. DR participated in the conceptualisation of the study and helped to draft the manuscript. RB participated to the conception of the analysis that was performed. AP realized the statistical analysis. All authors read and approved the final manuscript.

## Authors’ information

Mylaine Breton, PhD, is an assistant professor in Department of Community Health Science, Faculty of Medicine, University of Sherbrooke and she is a researcher in research in Research Center Charles-Lemoyne Hospital. Raynald Pineault, MD, PhD, is an emeritus professor in the Department of Health Administrator, Institut national de santé publique, Faculty of Medicine, University of Montréal. Jean-Frédéric Lévesque, MD, PhD, is the Chief Executive of the Bureau of Health Information in Australia. Danièle Roberge, PhD, is a associate professor in the Department of Community Health Science and she is a senior researcher in Research Center Charles-Lemoyne Hospital. Roxane Borgès Da Silva, PhD, is an assistant professor in Nursing Faculty, University of Montréal. Alexandre Prud’homme, Msc, is a research assistant, Institut national de santé publique.

## Pre-publication history

The pre-publication history for this paper can be accessed here:

http://www.biomedcentral.com/1472-6963/13/262/prepub

## Supplementary Material

Additional file 1 Details of comparison among different models of primary health care for collaborations within or outside local health network (LHNs). Click here for file

Additional file 2 Details of the comparison among clinics located in urban area and clinics located in rural area. Click here for file
