# Supplementary material for: Reforming healthcare systems on a locally integrated basis: is there a potential for increasing collaborations in primary healthcare?
Source: BMC Health Serv Res. 2013 Jul 8;13:262. doi: 10.1186/1472-6963-13-262 (PMC3750424; doi:10.1186/1472-6963-13-262)
Supplement: Additional file 1 — Details of comparison among different models of primary health care for collaborations within or outside local health network (LHNs). [file 1472-6963-13-262-S1.docx]

Appendix 1 details of comparison among different models of primary health care for collaborations within or outside local health network (LHNs)
