# Supplementary material for: Reforming healthcare systems on a locally integrated basis: is there a potential for increasing collaborations in primary healthcare?
Source: BMC Health Serv Res. 2013 Jul 8;13:262. doi: 10.1186/1472-6963-13-262 (PMC3750424; doi:10.1186/1472-6963-13-262)
Supplement: Additional file 2 — Details of the comparison among clinics located in urban area and clinics located in rural area. [file 1472-6963-13-262-S2.docx]

Appendix 2: Details of the comparison among clinics located in urban area and clinics located in rural area
